# Non-canonical Expression of Cardiac Troponin-T in Neuroendocrine Ethmoid Sinus Carcinoma Following Immune Checkpoint Blockade

**DOI:** 10.3389/fcvm.2019.00124

**Published:** 2019-08-23

**Authors:** Toshihiro Tsuruda, Yuichiro Sato, Kei Kajihara, Takayuki Kawabata, Yoko Kubuki, Soichi Komaki, Masao Kikuchi, Tetsunori Ishikawa, Tetsuya Tono, Kazuo Kitamura

**Affiliations:** ^1^Department of Internal Medicine, Circulatory and Body Fluid Regulation, Faculty of Medicine, University of Miyazaki, Miyazaki, Japan; ^2^Department of Diagnostic Pathology, Faculty of Medicine, University of Miyazaki, Miyazaki University Hospital, Miyazaki, Japan; ^3^Department of Otolaryngology, Head & Neck Surgery, Faculty of Medicine, University of Miyazaki, Miyazaki, Japan; ^4^Department of Gastroenterology and Hematology, Faculty of Medicine, University of Miyazaki, Miyazaki, Japan; ^5^Department of Cardiovascular Medicine, Miyazaki Prefectural Nobeoka Hospital, Miyazaki, Japan

**Keywords:** troponin, creatine kinase, MRI, echocardiogram, neuroendocrine tumor

## Abstract

We describe the case of a patient with neuroendocrine ethmoid sinus carcinoma, who exhibited markedly elevated levels of serum cardiac troponin-T and creatine kinase (CK)-MB isoenzyme without any symptom after the administration of nivolumab, immune checkpoint inhibitor. The repeated 12-leads-electrocardiogram did not show any changes in the ST-T segments or arrhythmias. The echocardiogram showed normal ranges of left ventricular contraction in the clinical course. Cardiac magnetic resonance imaging showed minimal myocardial edema and inflammation. Blood clots in the metastatic lesion of bone marrow aspirates exhibited positive staining for cardiac troponin-T and CK-MB in the cytoplasm and nucleoplasm of neoplastic cells. Although we did not perform a second cardiac magnetic resonance imaging and autopsy, we postulate that the attack of the neoplastic cells by the immune checkpoint inhibitor or the secretion from neoplastic cell-derived extracellular vesicles may have exacerbated the increase in concentrations of these molecules in the blood. Our case should warrant consideration a false-positive value of cardiac troponin-T and CK-MB can be obtained in cases with malignancy.

## Introduction

Patients with cancer can have high levels of different cardiovascular peptides (including troponin-T) prior to the initiation of anti-cancer therapy and alongside the presence of cardiac dysfunction (1), giving way to the hypothesis that the cancer could induce subclinical myocardial damage. In addition, neoplastic cell progression and cardiomyocyte survival shares common molecular signals, and the anti-cancer therapies result in cardiac toxicity (2). This suggests a close relationship between cancer and cardiovascular homeostasis, with the unmet medical need being to protect the heart from cancer and manage the adverse effects of anti-cancer therapy. Immune checkpoint inhibitors are a new class of anti-cancer drugs that interfere with the immune system, recognizing and targeting neoplastic cells (3). Wide-spread use of immune checkpoint inhibitors has resulted in immune-related adverse events, such as digestive and endocrine disorders, with myocarditis being one of the most serious complications (4, 5). The diagnosis of myocarditis is determined by the release of cardiac specific proteins/enzymes into the blood (6). However, it is hard to check the molecules if the patients are asymptomatic or show no signs of heart failure.

We describe the case of a patient with markedly elevated levels of serum cardiac troponin-T and creatine kinase (CK)-MB isoenzyme without any symptom after the administration of nivolumab, immune checkpoint inhibitor. We discuss the diagnostic workup of the immune checkpoint inhibitor-related myocarditis and non-canonical expressions of cardiac troponin-T and CK-MB isoenzyme in neoplasms.

## Case Presentation

**Figure 1:**
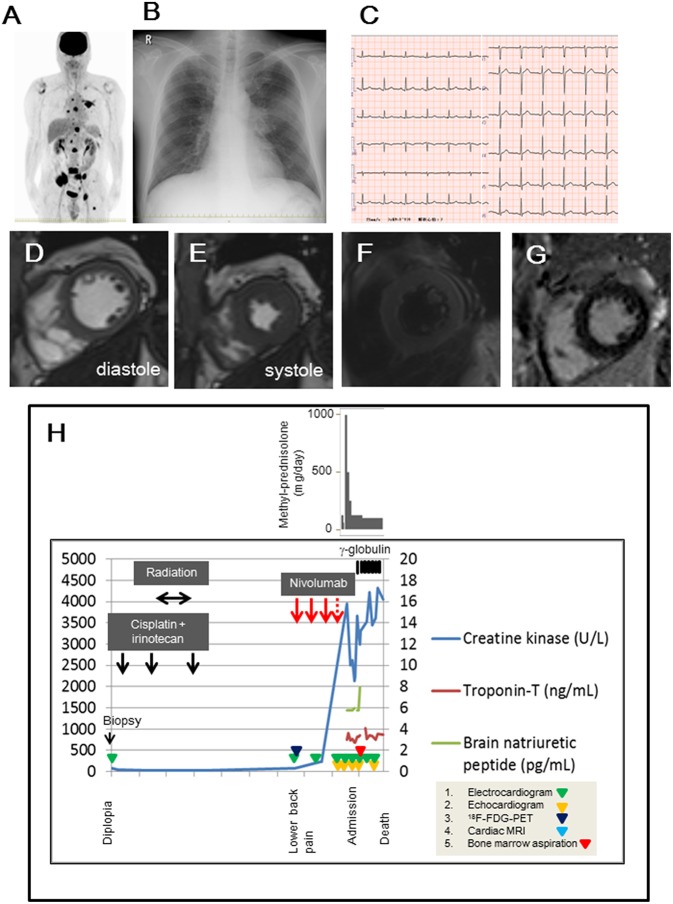
**(A)** ^18^F-fluorodeoxyglucose positron emission tomography (^18^F-FDG-PET) findings before nivolumab administration. **(B,C)** Chest radiograph and 12-leads-electrocardiogram obtained on the admission day. **(D,E)** Cardiac magnetic resonance imaging in diastole **(D)** and systole **(E)**, suggesting that global left ventricular function was not impaired. **(F)** The dark-blood sequence for non-enhanced T2-weighted image showed slight enhancement in the septal and lateral walls. **(G)** Delayed gadolinium-enhanced image showed minor enhancement in the mid-myocardial septal and inferior wall. **(H)** Examination and treatment of the clinical course. Electrocardiogram, echocardiogram, ^18^F-FDG-PET, cardiac magnetic resonance imaging, and bone marrow aspiration were performed on the indicated days in the panel.

A 47-year-old man with suspected myocarditis, due to nivolumab therapy was admitted to our hospital. He complained of diplopia 8-months prior to admission and was diagnosed with ethmoid sinus cancer (T4bN2bM0) at the referral hospital. The biopsy specimen showed positive staining for insulinoma-associated protein 1 (INSM1) or neural cell adhesion molecule 1 (CD56), but negative staining for nuclear protein in testis or synaptophysin, indicating neuroendocrine carcinoma. Systemic chemotherapy (cisplatin and irinotecan) and radiotherapy were administered. However, ^18^F-fluorodeoxyglucose-positron emission tomography scan suggested multiple bone metastases (Figure 1A). Nivolumab (3 mg/m^2^) was started and administered every 2 weeks. Although the fourth administration was scheduled, nivolumab was discontinued because of elevation in the levels of total CK (946 U/L; reference range, 30–200 U/L), CK-MB (484 IU/L; reference range, 0 to 12 IU/L), and cardiac troponin-T (1.25 ng/mL; reference range, <1 ng/mL) in the serum, which was evident at 16 days since the third nivolumab administration. The patient received methyl-prednisolone (1,000 mg/day) for 3 days, after which it tapered to 500 mg/day for 3 days, 250 mg/day for 3 days, and 125 mg/day for 3 days at the referral hospital. Thereafter, he developed lower back pain but no chest discomfort or palpitation.

**Figure 2:**
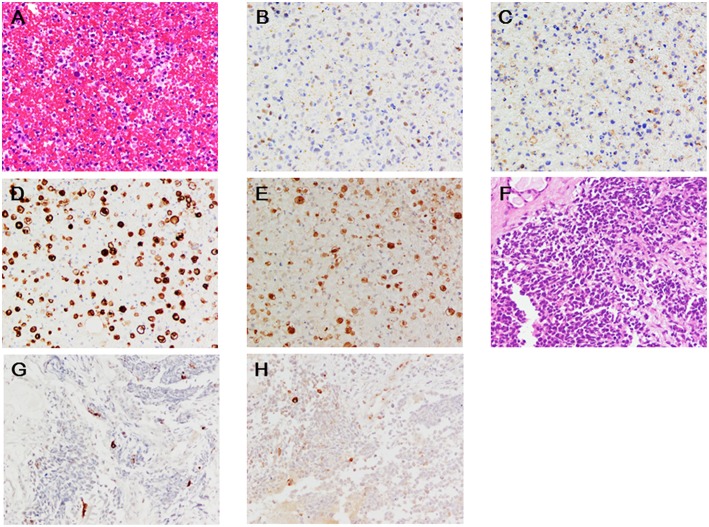
**(A–E)** Blood clot aspirates from the left side of the pelvis (**A**, hematoxylin-eosin; **B**, INSM1; **C**, CD56; **D**, troponin-T; **E**, CK-MB). **(F-H)** Primary lesion in the ethmoid sinus (**F**, hematoxylin-eosin; **G**, troponin-T; **H**, CK-MB). x200. Deparaffinized slide sections were heated to retrieve the antigen (citrate, pH 6.0 for CD56, Troponin-T, and CK-MB; ethylenediaminetetraacetic acid, pH 9.0 for INSM1). They were immersed in hydrogen peroxide to block the endogenous peroxidase activity. The slide sections were incubated with the following primary monoclonal antibodies; INSM1 (0.67 μg/mL, clone A-8, Santa Cruz Biotechnology, Inc.), CD56 (0.11 μg/mL, clone 1B6, Leica Biosystems Newcastle Ltd), cardiac troponin T (0.5 μg/mL, clone 13-11, ThermoFisher Scientific), and polyclonal antibody for CK-MB (2.5 μg/mL, ThermoFisher Scientific, catalog number PA5-28920). Then, the slide sections were incubated with horseradish peroxidase-polymer secondary antibody at room temperature. They were visualized with 0.05% 3, 3′-diaminobenzidine containing hydrogen peroxide, and counterstained with hematoxylin. The slides were scanned under the Olympus BX53F microscope (Olympus, Tokyo, Japan). Negative (IgG) and positive (human heart) slides were prepared for troponin-T and CK-MB staining (data not shown).

On examination, his temperature was 36.9°C, the blood pressure was 130/82 mmHg, the heart rate was 90 beats/min, and the respiratory rate was 17 breaths/min. His weight was 67 kg, height was 169.3 cm, and body mass index was 23.4. Heart sounds were normal, and murmurs were inaudible. Chest radiographs revealed a 49% cardio-thoracic ratio, and hilum in the left lung showed a swollen lesion (Figure 1B). The twelve-leads-electrocardiogram showed a normal sinus rhythm, normal axis deviation, and no change in the ST-T segments (Figure 1C). The transthoracic echocardiogram showed a 63% ejection fraction without regional wall motion abnormality in the left ventricle, and the thickness of the intraventricular septal wall and left ventricular posterior wall at diastole were 10 and 11 mm, respectively. The global longitudinal strain was −19.3%. Cardiac magnetic resonance imaging showed normal systolic contractility (Figures 1D,E) and minimal myocardial edema and inflammation (Figures 1F,G). Blood test findings showed an increased number of white- blood cells (18,800 /mm^3^; reference range, 3,300–8,600 /mm^3^: neutrophils, 90.3 %; reference range, 37–72 %, eosinophils, 0.1 %; reference range, 0.6–8.3%), but a decrease in the platelet cell count (70,000/mm^3^; reference range, 158,000–348,000/mm^3^). The levels of lactate dehydrogenase (8,695 U/L; reference range, 124–222 U/L), aspartate aminotransferase (157 U/L; reference range, 13–30 U/L), total CK (3,385 U/L; reference range, 59–248 U/L), MB isoenzyme (1,221 U/L; reference range, <12 U/L), and cardiac troponin-T (3.30 ng/mL; reference range, <0.1 ng/mL) were markedly elevated. Electrophoretic findings did not show the presence of the macro CK isoenzyme (CK-BB, 2%; CK-MB, 33%; CK-MM, 65%). The C-reactive protein level was 0.50 mg/dL (reference range, 0–0.14 mg/dL). The brain natriuretic peptide level was normal (<5.8 pg/mL; reference range, <18.4 pg/mL). We continued to administer 125 mg/day of methyl-prednisolone, and we added 5 g/day of γ-globulin for 7 days, but the patient persistently showed elevated levels of total CK and cardiac troponin-T within 1 month (Figure 1H). The twelve-leads-electrocardiogram was repeated 7 times after nivolumab administration, but it did not show any changes in the ST-T segments or arrhythmias. In fact, the echocardiogram was performed 5 times after nivolumab administration, and showed a normal range of left ventricular ejection fraction (58~63%) in the clinical course, despite elevated serum cardiac troponin-T and CK-MB isoenzyme levels. This finding raised the concern of whether cardiac troponin-T and CK-MB isoenzymes were released from the damaged myocardium into the blood because of nivolumab. Blood clots in the bone marrow aspirates obtained from the left side of the pelvis exhibited consistent immunohistological features of the primary lesion, which showed positive staining for INSM1 and CD56 (Figures 2A–C). These clots also showed positive staining for cardiac troponin-T (Figure 2D) and CK-MB (Figure 2E) in the cytoplasm and nucleoplasm of metastatic neoplastic cells. These molecules were also scattered in the primary lesion of the ethmoid sinus (Figures 2F–H). The patient died from progressive disease at 10 months after the initial presentation, and postmortem autopsy was not performed.

## Discussion

Our report shows that some neoplastic cells contain cardiac-specific proteins and enzymes, which are released into the blood stream, and this release mimics the characteristics of myocarditis when the immune checkpoint blockade has already been administered.

Elevations of cardiac troponin-T and CK-MB isoenzyme in the serum reflect myocardial damage—these levels are used as diagnostic markers for acute myocardial infarction (7). In particular, troponin level is superior to electrocardiographic/echocardiographic findings, or N-terminal pro-brain natriuretic peptide levels for detecting the immune therapy-associated myocarditis (8). However, our case should warrant consideration a false-positive value can be obtained in cases with malignancy. The CK-MB isoenzyme has been reported in metastatic lung cancer following the chemotherapy (9), and the elevation of CK-MB isoenzyme reflects the clinical course in rhabdomyomatous Wilms tumor (10). The metastatic form of human non-small cell lung cancer aberrantly expresses troponin-I (11). Small cell neuroendocrine carcinoma of nasal and paranasal cavities produces ectopic hormones, including antidiuretic hormone, parathyroid hormone, and serotonin (12). However, there is no report describing troponin and CK-MB isoenzyme in this type of neoplastic cells. In our case, both cardiac troponin-T and CK-MB immunoreactivity were scattered in the specimens of the primary site of neoplastic cells, but they were abundant in metastatic lesions. Cardiac troponin-T and CK-MB positive neoplastic cells are likely to be resistant to chemotherapy/immunotherapy. We postulate that the attack of the neoplastic cells by the immune checkpoint inhibitor or the secretion from neoplastic cell-derived extracellular vesicles (13) may have exacerbated the increase in concentrations of cardiac troponin-T and CK-MB isoenzyme levels in the blood. The functional roles of cardiac troponin-T and CK-MB isoenzyme in neoplastic cells remain unknown. Troponin subunits enhance the growth and migration of neoplastic cells (13). Adenosine diphosphate generated by CK produces energy for cellular functions, and stimulates the degradation of the extracellular matrix (14). We speculate that the accumulation of cardiac-specific proteins/enzymes may contribute to disease progression in a tumor environment. Further studies are necessary to clarify this finding.

The prevalence of immune checkpoint inhibitor-related myocarditis has been reported to be 0.09–1.14% (8), and the severity ranges from subclinical to lethal (15, 16). After considering the possible cardio-toxic effects of nivolumab, we discontinued nivolumab treatment. Despite methyl-prednisolone and γ-globulin administration, serum cardiac troponin-T and CK-MB isoenzyme levels remained high. Platelet numbers progressively decreased, and endomyocardial biopsy was contraindicated. Echocardiogram can help to detect early signs of cardiac dysfunction in diagnosing myocarditis and in a close follow up in patients with anti-cancer therapy (17, 18), and our patient demonstrated normal ranges of left ventricular ejection fraction and myocardial strain in the clinical course. Both echocardiographic findings and plasma brain natriuretic peptide concentration did not indicate the overt cardiac dysfunction. In addition, even if cardiac magnetic resonance imaging revealed the presence of minimal signs of myocardial edema and inflammation, it did not explain the elevated cardiac troponin-T and CK-MB isoenzyme levels. Therefore, it was a matter of debate whether nivolumab should be ceased or it should be continued before having any confirmations from an imaging test of myocarditis. It is also uncertain whether cardiac troponin-T presented by the neoplastic cells is recognized as the antigen of T-cells clone and whether this injures the myocardium following the immune checkpoint blockade (19). Evaluation of troponin-T expression prevalence in the neoplasm in cases of immune checkpoint inhibitor-related myocarditis might prove this hypothesis. We did not measure troponin-T level and perform any imaging tests, i.e., echocardiography and cardiac magnetic resonance imaging, prior to the nivolumab administration. Subsequent cardiac magnetic resonance imaging during the clinical course, and postmortem autopsy could have additive value in this case report.

In conclusion, this report shows the non-canonical expressions of cardiac troponin-T and CK-MB isoenzyme in neoplastic cells. Our experience highlights that the analysis of the patient following immune checkpoint blockade therapy should be carefully interpreted.

## Data Availability

The datasets generated for this study are available on request to the corresponding author.

## Ethics Statement

This study was approved by the Human Investigation Review Committee of the University of Miyazaki (No. 0-0511) and conformed with the principles outlined in the Declaration of Helsinki as revised in 2013. We state that, because the patient passed away, written informed consent was obtained from his mother, for the publication of this case report.

## Author Contributions

Four cardiologists (TTs, TI, KKi, and SK), a nephrologist (MK), three otolaryngologists (KKa, TK, and TTo), and one hematologist (YK) were involved in the treatment of the patient in our case report. One pathologist (YS) performed the histological analysis.

### Conflict of Interest Statement

The authors declare that the research was conducted in the absence of any commercial or financial relationships that could be construed as a potential conflict of interest.
